# The microneedles carrying cisplatin and IR820 to perform synergistic chemo-photodynamic therapy against breast cancer

**DOI:** 10.1186/s12951-020-00697-0

**Published:** 2020-10-19

**Authors:** Ji-jun Fu, Chu-wen Li, Yang Liu, Ming-yue Chen, Qiang Zhang, Xi-yong Yu, Bo Wu, Jie-xia Li, Ling-ran Du, Yuan-ye Dang, Dan Wu, Min-yan Wei, Zhi-qiang Lin, Xue-ping Lei

**Affiliations:** 1 The Key Laboratory of Molecular Target & Clinical Pharmacology, School of Pharmaceutical Sciences and the First Affiliated Hospital of Guangzhou Medical University, Guangzhou Medical University, Guangzhou, 511436 China; 2 Institute of Systems Biomedicine, Beijing Key Laboratory of Tumor Systems Biology, School of Basic Medical Sciences, Peking University Health Science Center, Beijing, 100191 China; 3 School of Pharmaceutical Sciences, Zhengzhou University, No.100, Kexue Avenue, Zhengzhou, 450001 China

**Keywords:** Microneedles (MN), Cisplatin (CDDP), IR820, Photodynamic therapy (PDT), Chemotherapy

## Abstract

**Backgrounds:**

Surgical resection and adjunct chemotherapy or radio-therapy has been applied for the therapy of superficial malignant tumor in clinics. Whereas, there are still some problems limit its clinical use, such as severe pains and side effect. Thus, it is urgent need to develop effective, minimally invasive and low toxicity therapy stagey for superficial malignant tumor. Topical drug administration such as microneedle patches shows the advantages of reduced systemic toxicity and nimble application and, as a result, a great potential to treat superficial tumors.

**Methods:**

In this study, microneedle (MN) patches were fabricated to deliver photosensitizer IR820 and chemotherapy agent cisplatin (CDDP) for synergistic chemo-photodynamic therapy against breast cancer.

**Results:**

The MN could be completely inserted into the skin and the compounds carrying tips could be embedded within the target issue for locoregional cancer treatment. The photodynamic therapeutic effects can be precisely controlled and switched on and off on demand simply by adjusting laser. The used base material vinylpyrrolidone—vinyl acetate copolymer (PVPVA) is soluble in both ethanol and water, facilitating the load of both water-soluble and water-insoluble drugs.

**Conclusions:**

Thus, the developed MN patch offers an effective, user-friendly, controllable and low-toxicity option for patients requiring long-term and repeated cancer treatments.

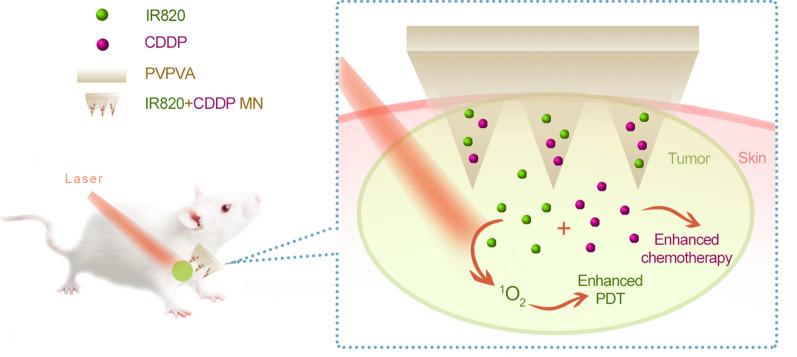

## Introduction

Superficial malignant tumor, including squamous cell carcinoma, malignant melanoma and breast cancer, is the most common tumors worldwide that with increasing morbidity and mortality [1]. Surgical resection and adjunct chemotherapy or radio-therapy has been applied for the therapy of superficial malignant tumor in clinics. Whereas, there are still some problems limit its clinic application. Surgery is quite invasive that requires long time to recover and remains high risks once the tumor tissues are removed incompletely. And patients with other diseases, such as heart failure, could not tolerate surgery. In addition, drug resistance result in unsatisfied efficacy of many chemotherapy drugs in the therapy of superficial malignant tumor, indicating by poor response duration and survival of patients [2, 3]. Thus, it is urgently needed to explore effective and low toxicity therapy stagey for superficial malignant tumor.

Previous studies showed that phototherapy combined with chemotherapy could obtain a perfect anti-tumor effect at lower drug dosages than conventional single treatments [4, 5]. In the case of photothermal therapy (PTT), with the near-infrared (NIR) laser exposure, the photothermal agents convert the light energy into the heat energy and, finally, the elevated environment temperature kills the tumor cells. In the case of photodynamic therapy (PDT), with the red laser or NIR exposure, the photosensitive agents convert oxygen into the reactive-oxygen species (ROS) to play the therapeutic effects. Compared to chemotherapy, phototherapy brings reduced systemic toxicity as elevated temperature or ROS will happen only in the unhealthy tissues exposed to laser. Both PTT and PDT are highly promising in treating malignant tumors. IR820, with similar chemical structure to indocyanine green (ICG), is a commonly used photosensitive agent. Under the 808 nm laser exposure, IR820 produces both light-heat transition and ROS generation. As a result, IR820 was reported to play PTT [6–11] and PDT [11–13] against cancer.

Systemic administration such as intravenous injection and traditional intra-tumor injection of drugs are usually used in phototherapy combined with chemotherapy. Systemic administration often need high dose to achieve a therapeutic dose in the tumor site, which would likely caused severe side effect [14]. As for traditional intra-tumor injection, relatively lower dosage is needed than systemic administration, whereas drug leakage to neighbor normal tissues often leading to side toxicity and lack of effective drug in tumor area. In addition, passive diffusion often result in a limited intratumoral drug amount in the tumor [15]. Therefore, exploring an effective, safe and minimally invasive transdermal approach for superficial malignant tumor therapy is needed.

Microneedle (MN) is needle arrays with micrometer dimensions and could effectively penetrate the skin barriers and then delivery into the tumor. Compared with the traditional needles, the MN is composed of tens of needles smaller than 1 mm. As a result, the MN is able to greatly reduce the injection pain and the skin injuries after repeated injections. As a result, the MN is anticipated to increase the patients’ compliance and the therapeutic effects. Nowadays, the MN has been employed to treat superficial malignant cancers [3, 4, 16–21]. The MN is reported to deliver the chemotherapy agents, such as CDDP [16] or doxorubicin [17]. Recently, several studies showed that MN patch could act as a novel synergetic system when combined with chemotherapy and phototherapy. Chen et al. developed a system consists of embeddable polycaprolactone MNs containing a photosensitive nanomaterial and DOX than could completely eradicated 4T1 tumors within 1 week [4]. Karls et al. prepared a MN patch loaded with indocyanine green (ICG) and DOX and obtained exciting anti-tumor effect with low toxicity [22]. And MN is able to carry antigens to stimulate anti-tumor immune responses [19, 20]. Despite the great achievements, the complicated processing technique is still a big challenge in the development of NM. For example, the commonly used base materials such as poly (L-lactide) is water-insoluble, the use of organic solvents restricts the encapsulation of water-soluble drugs. As water and ethanol are the most widely used solvents in the dosage forms, the base material which is soluble in both water and ethanol is preferred.

In this work, we employed vinylpyrrolidone—vinyl acetate copolymer (PVPVA) as the base material, loaded with CDDP and IR820 into the MN patches. The PVPVA is freely soluble in water and ethanol, and has been approved for clinical use as pharmaceutical excipient. And its water solution could easily reach the concentration of more than 20% while maintaining good fluidity. The MN could be completely inserted into the skin and the compounds carrying tips could be embedded within the target issue for locoreginal cancer treatment. The photodynamic therapeutic effects can be precisely controlled and switched on and off on demand simply by adjusting laser. With breast cancer as a model, this work is expected to provide an effective, rapid, secure and humanized MN for the therapy of superficial malignant tumor.

## Materials and methods

### Materials

CDDP, IR820, Hoechst 33342, methylthiazolyldiphenyl-tetrazolium bromide (MTT) and 1,3-diphenylisobenzofuran (DPBF) were purchased from Aladdin Company (Shanghai, China). PVPVA (trade name Kollidon VA64) was gifted by Basf Corporation (Shanghai, China). The polydimethylsiloxane (PDMS) MN molds were bought from Micropoint Technologies Pte Ltd (Singapore). The dihydroethidium (DHE) and the live/dead assay kit (Calcein AM/PI) were purchased from KeyGen Biotech (Nanjing, China). The total reactive oxygen species assay kit (DCFH-DA), the active caspase-3 assay kit (GreenNuc) and the TUNEL apoptosis assay kit were obtained from Beyotime Biotechnology (Shanghai, China). The anti-Ki67 and anti-cleaved caspase 3 primary antibodies were purchased from Servicebio Technology (Wuhan, China). Other regents were analytical grade.

### Preparation of the MN

PVPVA was dissolved in deionized water to make a final concentration of 20% (w/v). CDDP and IR820 were dissolved in deionized water to get a mixture solution, each concentration was 10 mg/mL. Then, equal volume of the drug solution was combined with the PVPVA solution. The MN patches were fabricated by a two-step centrifugation casting method. In the first centrifugation process, 30 µL of the above drugs containing PVPVA solution was added into the PDMS molds, centrifugated at 3000 rpm for 5 min. Then, the redundant liquid beyond the microneedle holes was discarded and, the molds were dried in a dry cabin away from light for 12 h. Afterwards, the second centrifugation was carried out. 200 µL of the 20% PVPVA solution was added into the PDMS molds, centrifugated at 3000 rpm for 2 min and, dried for another 12 h. The CDDP-IR820 MN patches were obtained after the demold, then they were stored in a dry cabin away from light. The CDDP MN, IR820 MN and blank MN patches were prepared accordingly, with one drug or both compounds absent.

### Characterization of the MN patches

The morphology of the blank MN, CDDP MN, IR820 MN and CDDP-IR820 MN patches were imaged by bright field microscope, fluorescence microscope (DMi8, Leica, Germany) and scanning electron microscope (SEM, JSM-6510, Shimadzu).

Dissolving one MN patch in 1 mL of deionized water, atomic absorption spectrophotometer (AAS) (ICE3000, Thermo Scientific, U. S. A.) and ultraviolet–visible (UV) spectrum (UV-2600, Shimadzu, Japan) were used to determine the concentration of IR820 and/or CDDP. The drug loading amount was calculated according to the following equation

1 $${\text{Drug loading amount }}(\mu {\text{g per patch}}) \, = {\text{ water solution concentration }}(\mu {\text{g}}/{\text{mL}})\times{\text{1 mL}}$$

As the total volume of the MN tips is 1.67 µL and the concentration of CDDP and IR820 in the PVPVA solution is 10 mg/mL, the encapsulation efficiency is calculated according to Eq. 2:

2 $${\text{Encapsulation efficiency }}\left( \% \right) \, = {\text{ drug loading amount }}/{ 16}.{7}\times{1}00\%$$

The mechanical features of the MN patches were evaluated by a texture analyzer (XTPlus, Stable Micro Systems, TA, U. K.). The instrument was equipped with 5 mm stainless cylinder. The pre-test speed, the test speed and the post-test speed were 0.1 mm/s. As the MN height was 800 µm, the maximum test distance was set to be 700 µm. All the tests were performed at room temperature and, repeated at least three times.

### Drug release from the MN patches

To accurately determine the drug release of the MN patch in vivo, the CDDP-IR820 MN patches were applied onto the mouse skin after hair remove. At different time points of 1, 2, 5, 10 min, the residual MN was pictured by bright field microscope and fluorescence microscope (DMi8, Leica, Germany). Finally, the residue MN patch was dissolved in 1 mL of deionized water and, the concentration of IR820 and CDDP was determined by UV spectrophotometer (UV-2600, Shimadzu, Japan) and AAS (ICE3000, Thermo Scientific, U. S. A.). The residual drug percentage was calculated according to the following equation:

3 $${\text{Residual drug percentage }}\left( \% \right) \, = {\text{ residual drug amount }}/{\text{ drug loading amount}}\times{1}00\%$$

### The assessment of ^1^O_2_ production

The ^1^O_2_ production was evaluated by employing DPBF decomposition reaction. In a typical experiment, the final concentration of DPBF was 100 µM and, the final concentration of IR820 and/or CDDP was 10 µg/mL. Dimethyl sulfoxide (DMSO) was used as the solvent. The samples were exposed to 808 nm laser at 100 mW/cm^2^. At different time intervals, the visible absorbance at 410 nm was measured (Epoch, Biotek, U. S. A.).

### MTT assay

The 4T1 mouse breast cancer cell line was used. The cells were cultured in Dulbecco’s modified Eagle’s medium with 10% fetal bovine serum and 1% penicillin – streptomycin. The cells were incubated in a humidified atmosphere containing 5% CO_2_ at 37 °C in a cell incubator.

MTT test was used to evaluate the anti-tumor effects of CDDP and/or IR820 in combination with NIR laser exposure. In a typical experiment, 1×10^4^ 4T1 cells were seeded in each well of 96-well plate. The cells were allowed to grow overnight for cell attachment. Then, CDDP was added to the media to get a serial final concentration of 0, 1, 2, 4, 10, 20 µg/mL. And, IR820 was added to the media to get a serial final concentration of 0, 1, 2, 4, 10, 20 µg/mL. 12 h later, the cells were exposed to 808-nm laser at 100 mW/cm^2^ for 5 min or not. After another 12 h, 10 µL of MTT solution (5 mg/mL) was added into each well. After incubating for 3 h, the media was discarded and, 100 µL of DMSO was added to each well to dissolve the formazan. Finally, the visible absorbance at 490 nm was measured (Epoch, Biotek, U. S. A.).

### The live/dead assay

In detail, 5×10^4^ 4T1 cells were seeded in each well of 24-well plate. After cell attachment, CDDP and/or IR820 was added into the media to get a final concentration of 2 and 10 µg/mL, respectively. After 12 h, the cells were exposed to 808-nm laser exposure at 100 mW/cm^2^ for 5 min or not. After further incubation for 0.5 h, the cells were co-stained with calcein AM and PI for 30 min. Then, the fluorescence microscope imaging was carried out (DMi8, Leica, Germany).

### The intracellular ROS detection

In detail, 5×10^4^ 4T1 cells were seeded in each well of 24-well plate. After cell attachment, CDDP and/or IR820 was added into the media to get a final concentration of 2 and 10 µg/mL, respectively. After 12 h, the cells were exposed to 808-nm laser exposure at 100 mW/cm^2^ for 5 min or not. After further incubation for 0.5 h, the fluorescence probe DCFH-DA or DHE was added to achieve a final concentration of 10 µM or 2 µM, respectively. After incubation for 30 min, ROS generation was immediately imaged by fluorescence microscope (DMi8, Leica, Germany). The excitation and emission wavelength were 450–490/515 nm for DCFH-DA and 510–550/575 nm for DHE.

### The caspase 3 activation analysis in vitro

In detail, 5 × 10^4^ 4T1 cells were seeded in each well of 24-well plate. After cell attachment, CDDP and/or IR820 was added into the media to get a final concentration of 2 and 10 µg/mL, respectively. After 12 h, the cells were exposed to 808-nm laser exposure at 100 mW/cm^2^ for 5 min or not. After further incubation for 0.5 h, the GreenNuc caspase 3 substrate was added to make a final concentration of 5 µM. After incubation for 30 min, the cells were washed by PBS twice and the nucleus was stained by Hoechst 33342. The fluorescence images were captured by fluorescence microscope (DMi8, Leica, Germany).

### In vivo therapeutic effects against breast cancer

The animal experiments were approved by the Ethic Committee of Guangzhou Medical University. The female Balb/c mice were used (6–8 weeks old) and, the mice were given free access to food and water.

6–8 weeks old female Balb/c mice were subcutaneously (*s.c.*) injected with 1 million of 4T1 cells in 200 µL of PBS to establish the tumor mice model. After the tumor reaching about 50 mm^3^, the mice were divided into four groups, the blank MN group, the CDDP MN group, the IR820 MN group and the CDDP-IR820 MN group. The mice were treated by the MN patches every other day (the dose of CDDP and/or IR820 was 15 µg per mouse) and, four treatments in total. The MN patches were applied onto the skin surrounding the tumors for 10 min to guarantee complete dissolution of the MN. 8 h after the MN application, the tumor sites were exposed to 808-nm laser at 100 mW/cm^2^ for 10 min. The tumor sizes and body weights were measured throughout the experiment and, the tumor volume was calculated according to the equation: volume = length×width^2^/2. At the end of the experiment, the mice were sacrificed and, the blood samples were collected and assayed and, the main organs of heart, liver, spleen, lung, kidney were sliced and H & E stained to reflect the systemic toxicity of the treatments. The skin tissues where the MN patches applied were also collected and H & E stained to assess the local toxicity. The tumor tissues were weighed and pictured. In addition, the TUNEL apoptosis, the Ki67 and the cleaved-caspase 3 expression of the tumor tissues were determined by immunohistochemistry (IHC).

### Statistic analysis

All values were expressed as mean ± standard deviation (SD). All comparisons were performed by the two-tailed Student's *t* test. A *P* value less than 0.05 was taken as statistically significant and a *P* value less than 0.01 was considered to be highly significant.

## Results

### Fabrication and characterization of the MN patches

Two-step casting method was used to prepare the MN patches. Firstly, the drugs carrying mixture solution was added to the mold to form the drugs-loading layer. Secondly, the drug-free PVPVA solution was added to construct the basal layer. Hyaluronic acid (HA), hydroxyproply methylcellulose (HPMC) and carboxymethylcellulose (CMC) are often used as the base material of the MN patches. In this study, PVPVA was employed as the base material. As a commercially available pharmaceutical excipient, there is no safety concerns. Moreover, PVPVA is soluble in both water and organic solvents, such as ethanol. The PVPVA solution with its concentration less than 20% (w/v) exhibits satisfactory fluidity to facilitate the MN construction. These features assigned PVPVA an ideal base material of the MN patches for both hydrophilic and hydrophobic drugs.

**Fig. 1:**
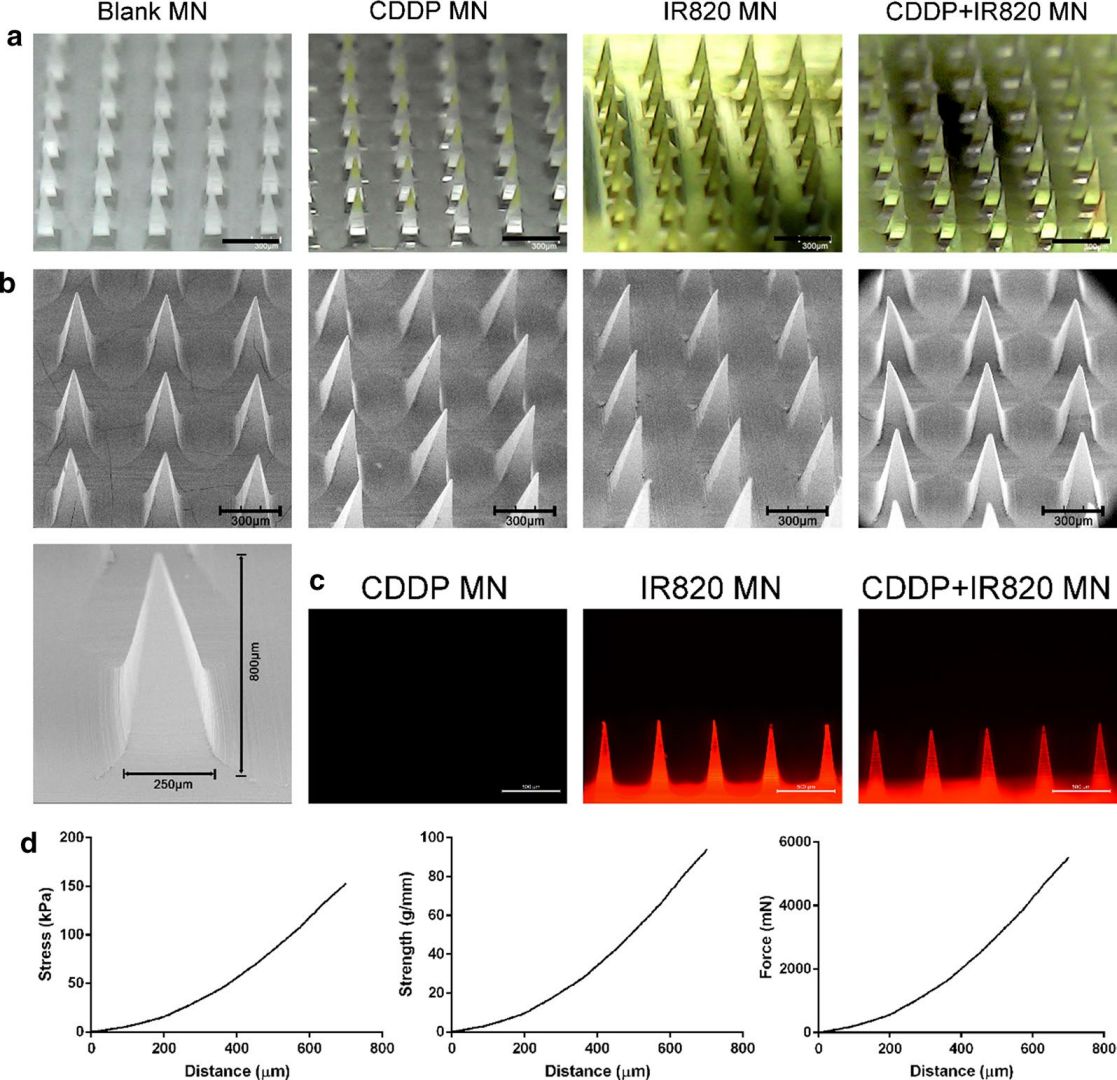
Morphology and mechanical features of the MN patches, **a** The bright field images, the scale bar represents 600 µm; **b** The SEM images, the scale bar represents 300 µm; **c** The fluorescence images, the scale bar represents 500 µm; **d** The stress—distance, strength—distance and force—distance profile of the MN patches

As shown in Fig. 1, the MN patch with 100 tips (10×10 tips square-arrayed in a 8 × 8 mm^2^ region) was fabricated. The dimensions of every tip were designed as a pyramid shape with a height of 800 µm and a base of 250 µm. The bright field microscope, SEM and the fluorescence microscope images of the four types of the MN patches were obtained. The morphology of the four kinds of tips was identical to each other. Each tip was in a pyramid shape and, the CDDP and/or IR820 encapsulation does not significantly influence the tip morphology. In addition, as shown in Fig. 1c, the IR820 and CDDP-IR820 MN patches could emit red fluorescence.

The total theoretical volume of MN tips calculated from the geometries (100 pyramid tips; base: 250 µm; height: 800 µm) was 1.67 µL. The dose of CDDP and/or IR820 packaged in each MN patch was determined to be about 15 µg. As the theoretical dose is 16.7 µg, the encapsulation efficiency was calculated to be 89.8%.

The mechanical features of the MN patches were shown in Fig. 1d. Along with the compression process, the stress, strength and force increases gradually. All the three profiles show a smooth and increasing trend, and, there was no breakpoint. The increasing curves indicated that the MN tips did not break throughout the test, even though the tips were compressed to only 100 µm height. The results implied that the MN tips had good ductility and, were not easy to be destroyed. In detail, at the end of the compression test, the stress, strength and force increased to 147.4 ± 20.4 kPa, 90.2 ± 12.5 g/mm, 5307.5 ± 733.4 mN, respectively. The data mean that the MN tips will be compressed to 100 µm height after putting counterweight of 557 g on a single MN patch, and, the tips will not break.

### Drug release from the MN patches

**Fig. 2:**
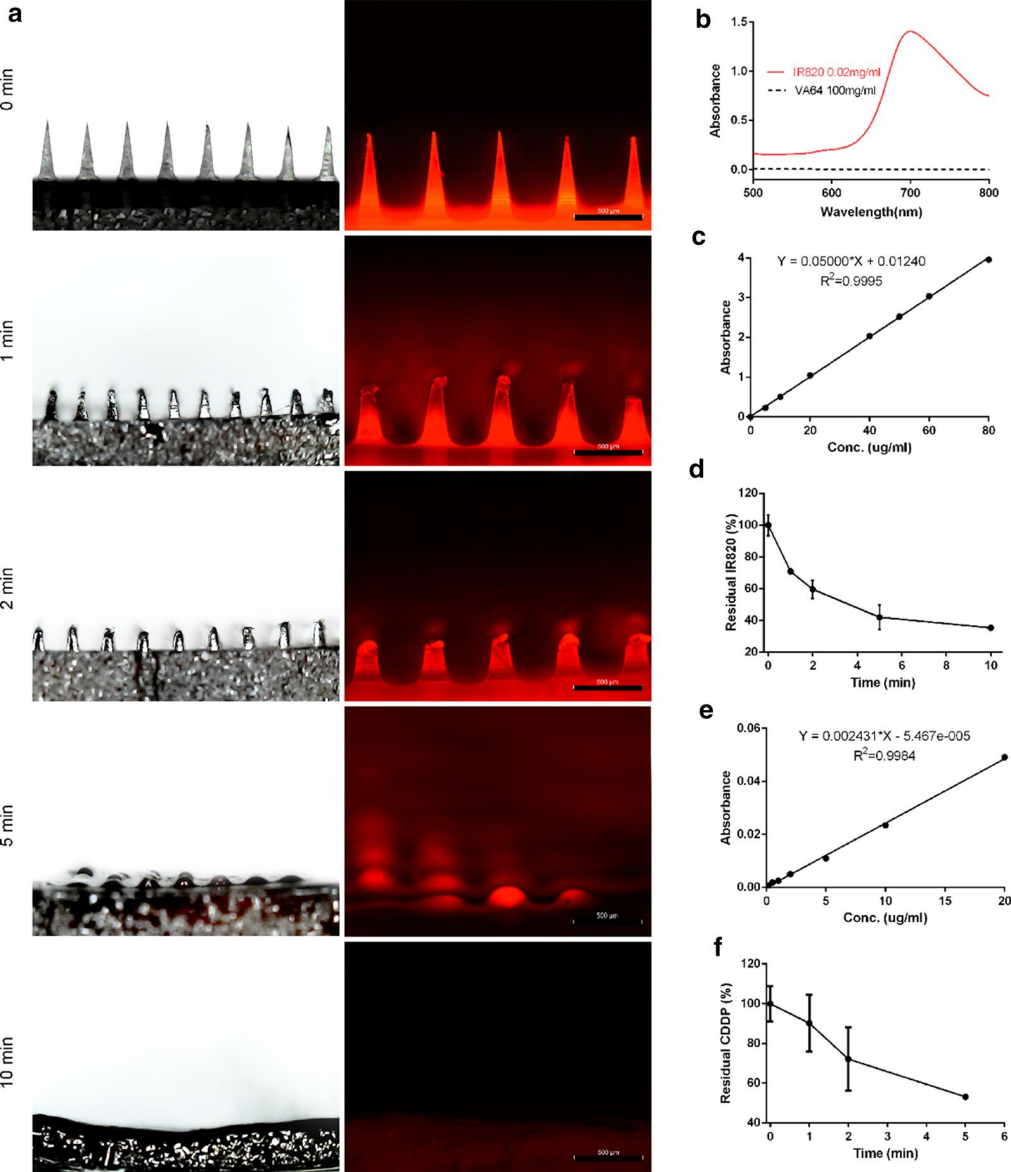
**a** The bright field and fluorescence microscope images of the MN patches after in vivo application on the mouse skin, the scale bar represents 500 µm; **b** The visible absorption spectrum of PVPVA and IR820; **c** The calibration curve of IR820 by measuring the visible absorbance intensity at 700 nm; **d** The residual percentage of IR820 in the MN patch after in vivo application on the mouse skin; **e** The calibration curve of CDDP by measuring the atomic absorbance intensity at 265.9 nm; **f** The residual percentage of CDDP in the MN patch after in vivo application on the mouse skin

The morphologies of the MN patches prior to and after penetrating the mouse skin were examined by the bright field and fluorescence microscope. As shown in Fig. 2a, the morphologies before and after the 10 min application of the MN patch on the mouse skin changed greatly. In horizontal views, pyramid-shaped MN tips nearly dissolved completely after the application. The experimental analysis of the actual drug released from the MN patches was performed. The base material PVPVA has negligible influence on the quantification of IR820 (Fig. 2b). The concentration of IR820 and/or CDDP residues in the MN patch was determined by the calibration curve which was established by measuring the visible absorbance intensity of IR820 at 700 nm (Fig. 2c, d) and the atomic absorbance intensity of Pt element at 265.9 nm (Fig. 2e, f). The quantitative analysis of in vivo application on mouse skin indicated that about 50% of CDDP and IR820 released after 5-min application. It means that nearly half of the compounds would not be uptake into the body. The phenomenon can be attributed to the fusion of the drugs from the tips to the basal layer during the drying of the MN patches.

### Singlet oxygen detection

**Fig. 3:**
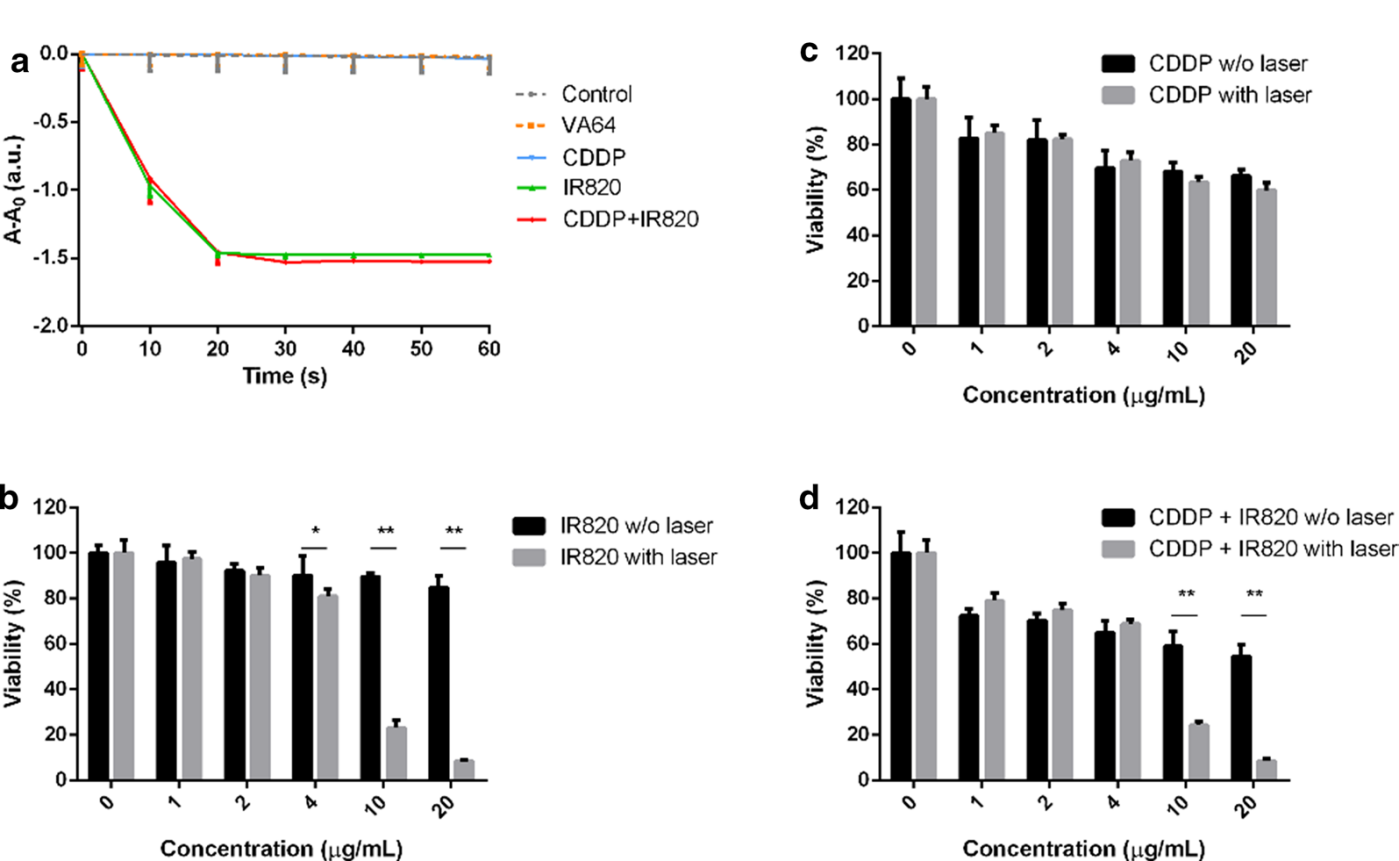
**a** The DPBF decomposition reaction upon laser irradiation; **c** The cytotoxicity of CDDP to 4T1 breast cancer cells; **b** The viability of 4T1 cells after the treatment of IR820 with or without laser irradiation; **d** The viability of 4T1 cells after the treatment of CDDP and IR820 with or without laser irradiation (*Indicates *p* < 0.05, **Means *p* < 0.01)

The singlet oxygen (^1^O_2_) generation capacity of IR820 upon near-infrared irradiation using DPBF as a probe was investigated. ^1^O_2_ generation was inversely proportional to the absorption of DPBF at the maximum absorption wavelength 410 nm. As shown in Fig. 3a, DPBF displayed a nearly constant absorption throughout the experiment, which ensured the reliability of this test. When exposed to 808 nm light, the absorption of DPBF in IR820 alone group and CDDP and IR820 mixture group quickly decreased in the first 20 s, which was attributed to the direct bleaching of ^1^O_2_ produced by IR820. IR820 and CDDP mixture exhibited a comparable DPBF-consuming rate to IR820 alone, it suggested the combined use of CDDP and IR820 would not compromise the photosensitizing ability of IR820. CDDP or PVPVA alone did not have any photosensitizing effect. After irradiation for 10 s and 20 s, the ^1^O_2_ generation percentage of IR820 alone and IR820 and CDDP mixture achieved 65% and nearly 100%, respectively.

### The biocompatibility and cytotoxicity in vitro

The promising photodynamic properties of IR820 inspired us to investigate the ability of IR820 for in vitro and in vivo anticancer treatment. Firstly, the compatibility of IR820 in cancer cells was investigated using MTT assay. After 24 h treatment, IR820 did not show significant toxicity toward 4T1 cell line (Fig. 3b). When its concentration reached 20 µg/mL, the survival rate was about 85%. Thus, this result suggest that IR820 has acceptable in vitro safety profiles and widen up possibility for its biomedical applications. Figure 3b also shows the cell viability after incubated with increasing concentration IR820 (0–20 µg/mL) upon NIR irradiation (808 nm, 100 mW/cm^2^). It should be mentioned that 100 mW/cm^2^ was particularly chosen to explore solely the photo—photodynamic effect of IR820. As shown in Fig. 3b a laser power density of 100 mW/cm^2^ did not induce obvious cell death to the PBS treated cells. And, under NIR irradiation, the cytotoxicity increased dramatically with the increase in IR820 concentration. In detail, IR820 with the concentrations of 4, 10, 20 µg/mL upon NIR irradiation resulted in cell viability of about 81, 23 and 8%, respectively. Moreover, the cell viability with and without laser exposure produced significantly statistic difference (*p* < 0.05). Figure 3c shows the cytotoxicity of CDDP alone. CDDP exhibited cytotoxic effects in a dose—dependent manner. It was obvious that the increased drug concentration produced decreased cell viability, with about 30% of cell death within 24 h at a CDDP dose of 4 µg/mL. As shown in Fig. 3d, compared to single PDT or chemotherapy, the synergistic PDT and chemotherapy produced enhanced cytotoxicity. The cell viability of 4T1 cells reduced to about 55% at the maximum concentration of CDDP and IR820 without NIR irradiation, compared to over 85% while treated by IR820 alone. Notably, the survival rate of the 4T1 cells irradiated with NIR was statistically lower than that of those without NIR irradiation, indicating laser irradiation improved the anticancer activity and further demonstrating the photodynamic effects of IR820.

**Fig. 4:**
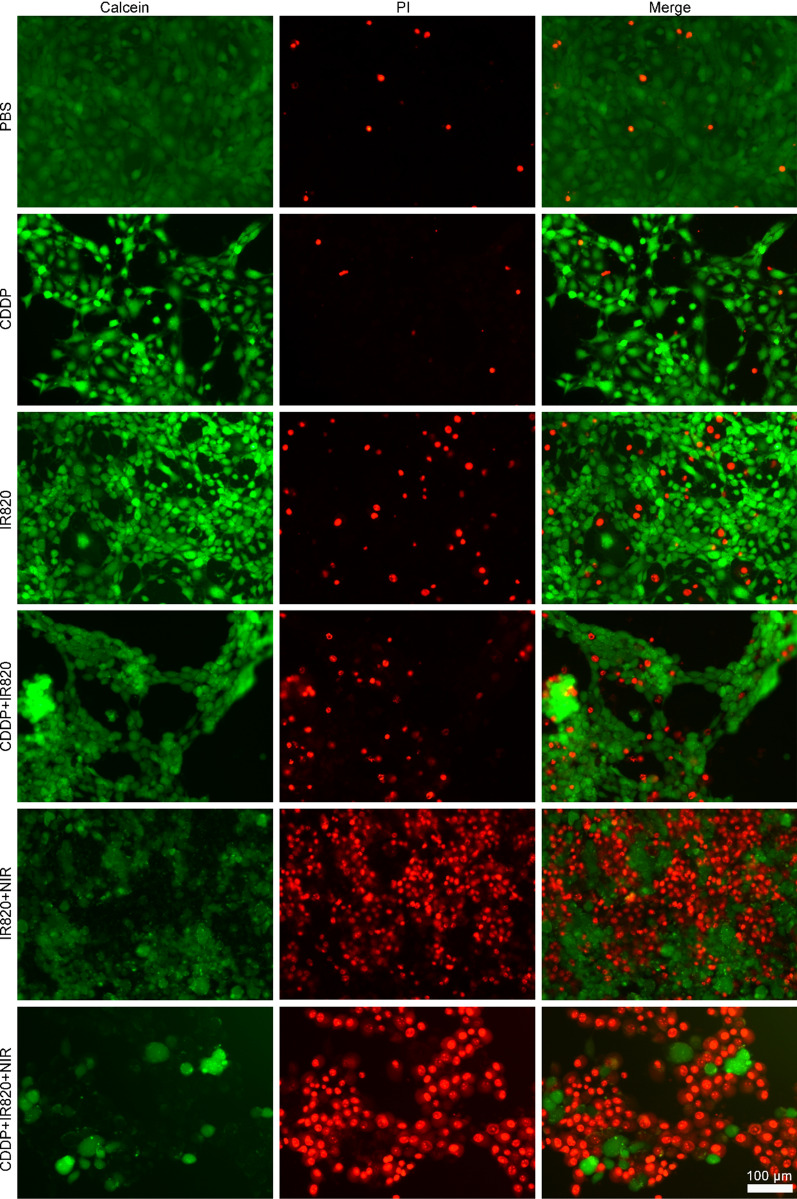
The live/dead assay of the 4T1 cells (the green fluorescence stands for living cells and the red fluorescence represents dead cells, the scale bar represents 100 μm)

To further visually evaluate the combinatorial anticancer ability of chemotherapy and PDT, 4T1 cells with different treatments were dyed with calcein AM and PI to distinguish the live and dead cells. Figure 4 indicated that more dead cells were observed in CDDP and IR820-treated group plus NIR irradiation than those of other groups. Notably, the combined use of CDDP and IR820 plus NIR irradiation almost entirely wiped out the 4T1 cells. As controls, neither PBS treatment nor IR820 alone in dark caused noticeable cell death. IR820 plus NIR irradiation group and CDDP groups without or with IR820 in dark all produced obvious cell death. It should be mentioned that dead cells might detached from the plate and, finally, would not be stained by PI. As a result, the red fluorescence alone could not indicate all the dead cells. In this case, the decrease in the green fluorescence could also indicate the cell loss. This phenomenon was observed in the CDDP groups, there was few PI positive cells, but the decrease in the calcein AM positive cell numbers indicated a great cell death.

### The intracellular total ROS detection

**Fig. 5:**
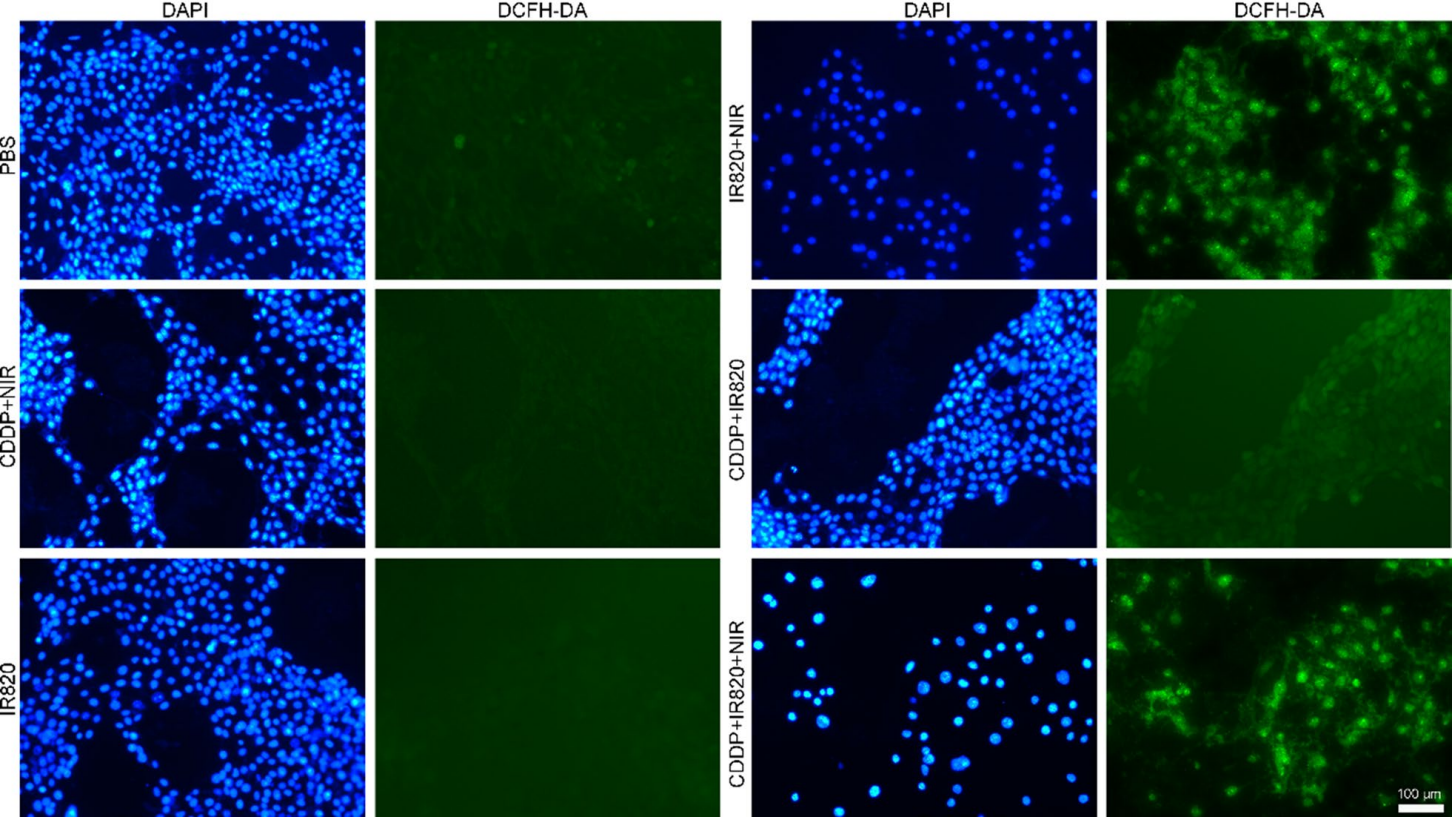
The intracellular total ROS generation detected by DCFH-DA (the blue fluorescence stands for nucleus and the green fluorescence represents ROS, the scale bar represents 100 μm)

**Fig. 6:**
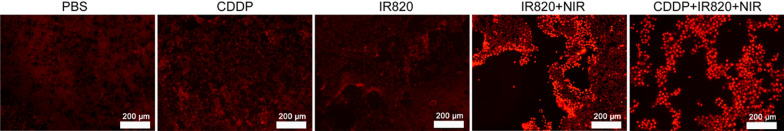
The intracellular ROS detected by DHE (the scale bar represents 200 μm)

To verify whether the PDT was activated under NIR exposure in the IR820 induced cell death, the total ROS generation in 4T1 cancer cells was assessed using DCFH-DA, a ROS-detecting fluorescent probe. DCFH-DA could be oxidized to 2′, 7′ – dichlorofluorescein emitting green fluorescence in the presence of ROS. As shown in Fig. 5, the PBS, CDDP, CDDP plus 808-nm light, IR820 or CDDP plus IR820 treated cells show weak green fluorescence, while IR820 plus 808-nm laser or CDDP and IR820 mixture plus 808-nm light triggered much stronger intracellular green fluorescence due to more activated DCFH-DA, indicating a high level of intracellular total ROS production. In a word, no or little ROS was generated in the cells without IR820, regardless whether NIR irradiation is performed. The combined use of IR820 and NIR irradiation promised pronounced ROS generation, which demonstrated the obvious photodynamic effect of IR820 in 4T1 cells. Additionally, the ROS production was further detected by the fluorescence of DHE. DHE is a ROS probe that has been widely used for the detection of ROS in the cells. DHE can penetrate cell membrane and be oxidized by ROS, leading to the formation of ethidium. Ethidium can imbed with the DNA, following with red fluorescence observed in nucleus. Thus, the nucleus fluorescence indicates the generation of ROS. And the fluorescence intensity is positively correlated with the extent of ROS generation. As shown in Fig. 6, groups received PBS, CDDP alone or IR820 alone in dark exhibited almost no red fluorescence in the nucleus, implying no ROS production in these groups. However, 4T1 cells treated with IR820 or CDDP and IR820 mixture upon NIR irradiation displayed strong red fluorescence in the nucleus, demonstrating generation of ROS in these treatments receiving laser irradiation.

### The caspase 3 activation

**Fig. 7:**
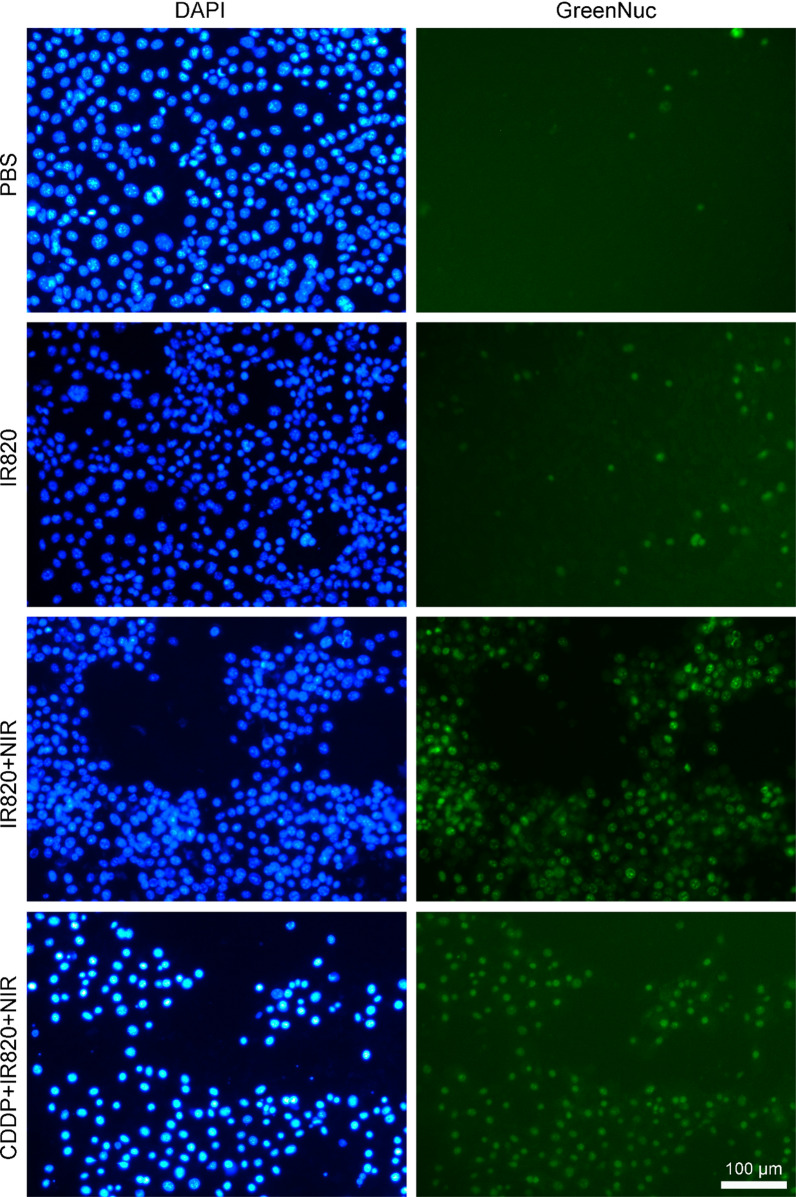
The caspase 3 activation assay. The blue fluorescence stands for nucleus and, the green fluorescence indicates the activation of caspase 3 and the cell apoptosis (the scale bar represents 100 µm)

To evaluate the cell apoptosis, the regent GreenNuc which could indicate the caspase 3 activation was employed. Caspase 3 activation is an important indicator of cell apoptosis. The GreenNuc regent contains green fluorescence dye labeled peptides. The green fluorescence dye is inclined to bind to DNA in the nucleus. However, the highly negatively charged DEVD sequence in the peptides retards the binding of the dye and the nucleus. The active caspase 3 could recognize the DEVD sequence and release the dye. Then, the free dye binds to the nucleus and emits the green fluorescence. In a word, the nucleus emitting green fluorescence indicates caspase 3 activation and cell apoptosis. As shown in Fig. 7, after incubation with IR820 in dark for 24 h, comparable to the PBS control, only a few green fluorescence positive nucleus were observed, demonstrating the low activation of caspase 3 and further indicating the low toxicity and good biocompatibility of the photosensitizer. However, a remarkable increase in the green fluorescence positive nucleus was found in the IR820 treated cells upon NIR exposure, implying the photodynamic effects of IR820. The CDDP – IR820 group irradiated by the 808 nm laser exhibited the best killing effect on 4T1 tumor cells with nearly all the nucleus emitting green fluorescence, suggesting the advantages of synergistic PDT and chemotherapy.

### The therapeutic effects of the MN patches in vivo

**Fig. 8:**
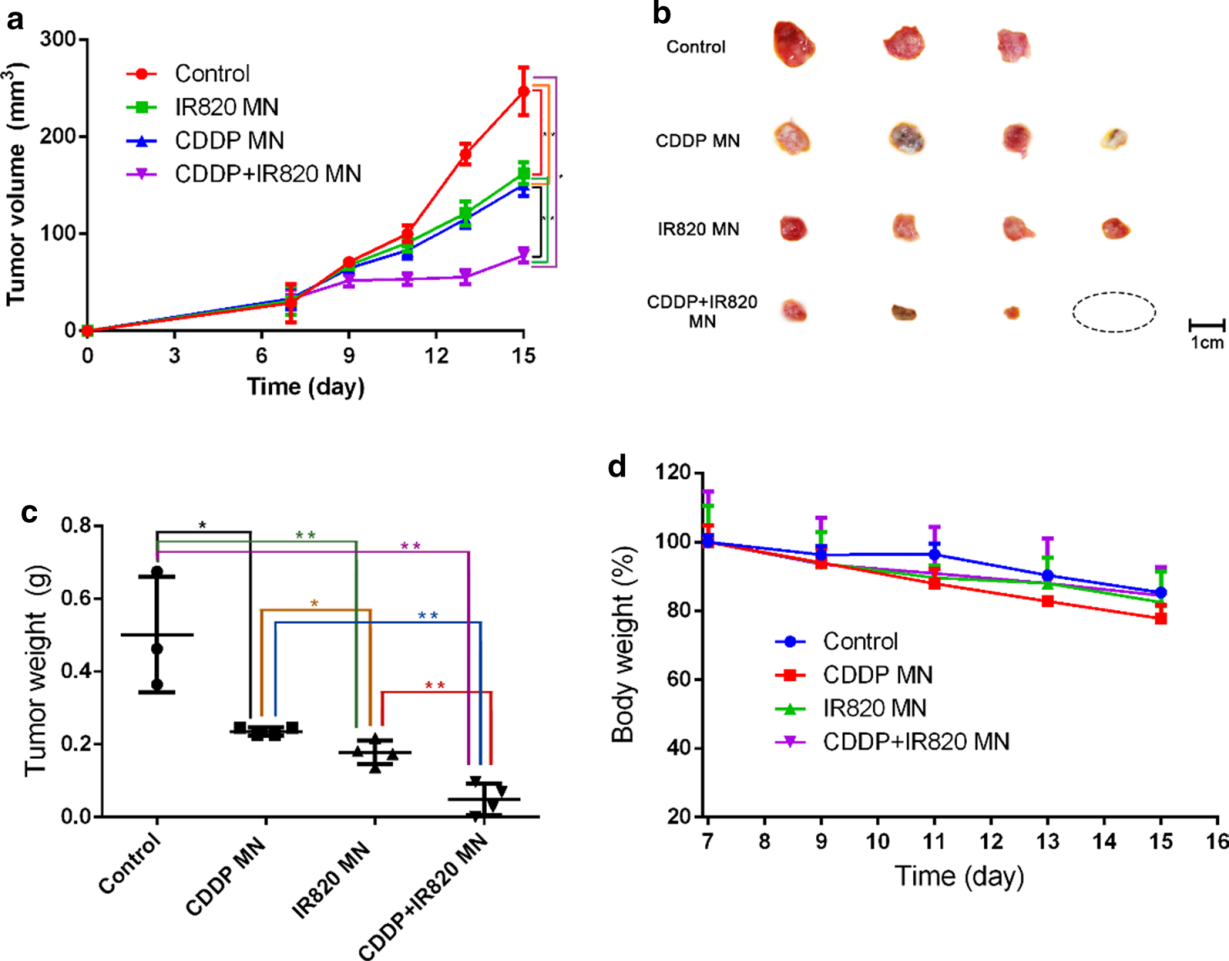
The therapeutic effects of the MN patches against breast cancer (*Represents statistic difference, *p* < 0.05). **a** The tumor growth profiles; **b** Pictures of the tumor samples after the treatment; **c** The statistic analysis of the tumor weights after the treatment (*Indicates *p* < 0.05, **Means *p* < 0.01); **d** Mice body weights throughout the treatment process

As ROS generation and the synergistic anticancer effects of CDDP and IR820 has been investigated in the cellular level, the synergistic PDT/chemotherapy treatment by the MN patches was further explored on 4T1 tumor-bearing mice. After peritumoral application of the MN patches, followed with laser irradiation, the tumor volume and animal body weight were recorded every other day (Fig. 8a, d). As shown in Fig. 8a, compared with other MN patches, mice treated with the blank MN patches with NIR irradiation (red curve) did not exhibit apparent tumor inhibition, with the tumor volume increasing rapidly and eventually reaching about 8.6-fold compared with that at the first treatment. Mice treated with CDDP MN (blue curve) or IR820 MN patches (green curve) upon NIR light displayed partial tumor inhibitory effect, and the tumor volumes eventually reached about 4.5-fold and 5.2-fold compared with that at the first treatment. Compared with other groups, the mice treated with CDDP-IR820 MN patches (purple curve) exhibited significantly delayed tumor growth, with the tumor volume eventually reaching about 2.4-fold compared with that at the first treatment. The tumors of this group were the smallest and sometimes even disappeared. At the end of the experiment, the tumor tissues were isolated, pictured and weighed. Pictures of the tumors (Fig. 8b) and the statistic analysis of tumor weights (Fig. 8c) were in accordance with tumor volumes. As shown in Fig. 8b, the CDDP MN and IR820 MN groups displayed moderate tumor growth inhibition. The CDDP-IR820 MN group shows the most obvious tumor growth inhibition, with one complete tumor regression. The tumor weights of different groups at the end of the experiment is shown in Fig. 8c. Statistically, the tumor weights of the CDDP MN (0.24 ± 0.01 g), IR820 MN (0.18 ± 0.03 g) and CDDP-IR820 MN (0.05 ± 0.04 g) groups was significantly smaller than the blank MN group (0.50 ± 0.16 g) and, the tumor weights of the CDDP-IR820 MN group was significantly smaller than the CDDP MN and IR820 MN group. The CDDP MN and IR820 MN group shows a moderate tumor inhibition ratio of 52.0% and 64.0%, and the CDDP-IR820 MN group shows a higher tumor inhibition ratio of 90.0%. It is worth noting that the tumor changes reflected by the tumor growth profiles and the final tumor weights are not exactly the same, that’s because certain amount of error exists in measuring the tumor volume due to the subjective judge and the disturbance of the skin. The excellent in vivo therapeutic effect of CDDP-IR820 MN patches against tumors was well correlated with the in vitro cytotoxicity data. The above results indicated the superior anticancer effects of CDDP-IR820 MN patches and the advantages of synergistic PDT/chemotherapy.

### The cell apoptosis and proliferation in the MN patches treated tumors

**Fig. 9:**
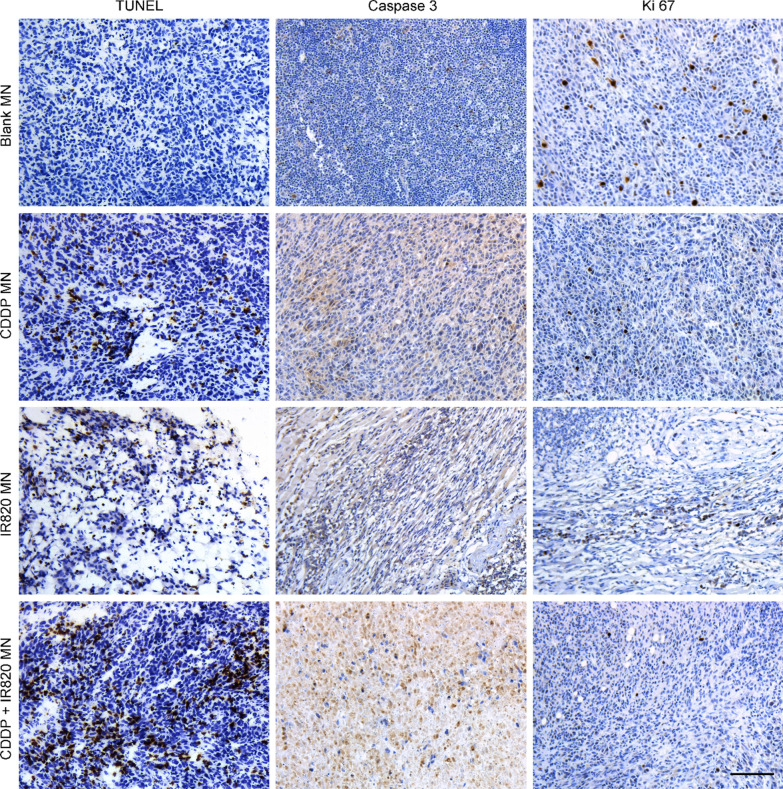
The histological analysis of the MN patches treated tumor samples, including TUNEL assay and the expression of cleaved-caspase 3 and Ki67 (the scale bar represents 100 µm)

To further assess the synergistic PDT/chemotherapy of CDDP-IR820 MN patches upon laser irradiation on 4T1 tumor-bearing mice, the tumor tissues were isolated and stained for histological analysis. Cell proliferation and apoptosis in the tumor tissues were analyzed through TUNEL staining, cleaved-caspase 3 and Ki67 assay (Fig. 9). Ki67 staining means cell proliferation, cleaved-caspase 3 and TUNEL staining implies cell apoptosis. As shown, little cell apoptosis was observed in tumors of the blank MN patches treated mice. And, tumors treated with CDDP MN, IR820 MN and CDDP-IR820 MN exhibited an obvious level of cell apoptosis. In detail, the CDDP-IR820 MN treatment induced the highest level of cell apoptosis and lowest rate of cell proliferation. The tumors treated with blank MN patches displayed the lowest rate of cell apoptosis and the highest rate of cell proliferation. Compared with the blank MN patches treatment, the IR820 MN or CDDP MN patches treated tumors exhibited enhanced cell apoptosis and reduced cell proliferation.

### The systemic toxicity of the MN patches treatments

In vivo toxicity was explored by conventional procedures. Figure 8d shows no obvious reduction in animal body weight throughout the treatment period, which proves the treatment is highly safe. Additional file 1. Fig. S1 shows H & E stained main organ sections after the MN patches treatment. As shown, the main organs did not show pathological changes in the cellular morphology or inflammatory infiltrates. Both the glomerulus structure in kidney and the hepatocytes in liver were found to be normal, pulmonary fibrosis was not observed in lung and, necrosis was not found in all the histological samples. In a word, histological examination of major organs from all the groups revealed no abnormality. The nonobvious abnormality of the blood samples from the differently treated mice further demonstrated excellent compatibility and negligible toxicity of the treatment (Additional file 1. Fig. S2). Additional file 1. Fig. S3 further shows that the skin tissues after the application of MN patches and/or 808 nm laser displayed normal cellular morphology and hair follicle structure, without obvious pathological changes. It proves that the synergistic chemotherapy and PDT through CDDP and IR820 in the MN patches has no safety concerns.

## Conclusions

In this work, MN patches loading CDDP and IR820 was prepared to perform chemo -photodynamic therapy against breast cancer. The MN patch has 100 tips in a 8×8 mm^2^ region. The two drugs could release into the body in five minutes after the application of the MN patch on the skin. IR820 was able to generate singlet oxygen, superoxide anion and other kinds of ROS upon laser irradiation in vitro and in cancer cells. The combination of CDDP and IR820 induced obviously intracellular ROS generation, caspase 3 activation and cell death. The CDDP—IR820 MN patches exhibited profound tumor growth inhibition in the aspects of enhanced cell apoptosis and reduced cell proliferation. The results implied the great advantages of synergistic chemotherapy and PDT through the MN patches. In addition, this treatment is featured with low cytotoxicity and good biocompatibility.

## Supplementary information

**Additional file 1:** **Figure S1.** H & E staining of the main organs after the MN patches treatment (the scale bar represents 200 µm). **Figure S2.** Blood analysis of the MN patches treated mice. **Figure S3.** H & E images of the skin tissues after the MN and/or 808 nm laser treatment (the scale bar represents 100 µm).

## Data Availability

All data used to generate these results is available in the main text.
