# Supplementary material for: The microneedles carrying cisplatin and IR820 to perform synergistic chemo-photodynamic therapy against breast cancer
Source: J Nanobiotechnology. 2020 Oct 19;18:146. doi: 10.1186/s12951-020-00697-0 (PMC7574214; doi:10.1186/s12951-020-00697-0)
Supplement: Supplementary file 1 — Additional file 1: Figure S1. H & E staining of the main organs after the MN patches treatment (the scale bar represents 200 µm). Figure S2. Blood analysis of the MN patches treated mice. Figure S3. H & E images of the skin tissues after the MN and/or 808 nm laser treatment (the scale bar represents 100 µm). [file 12951_2020_697_MOESM1_ESM.docx]

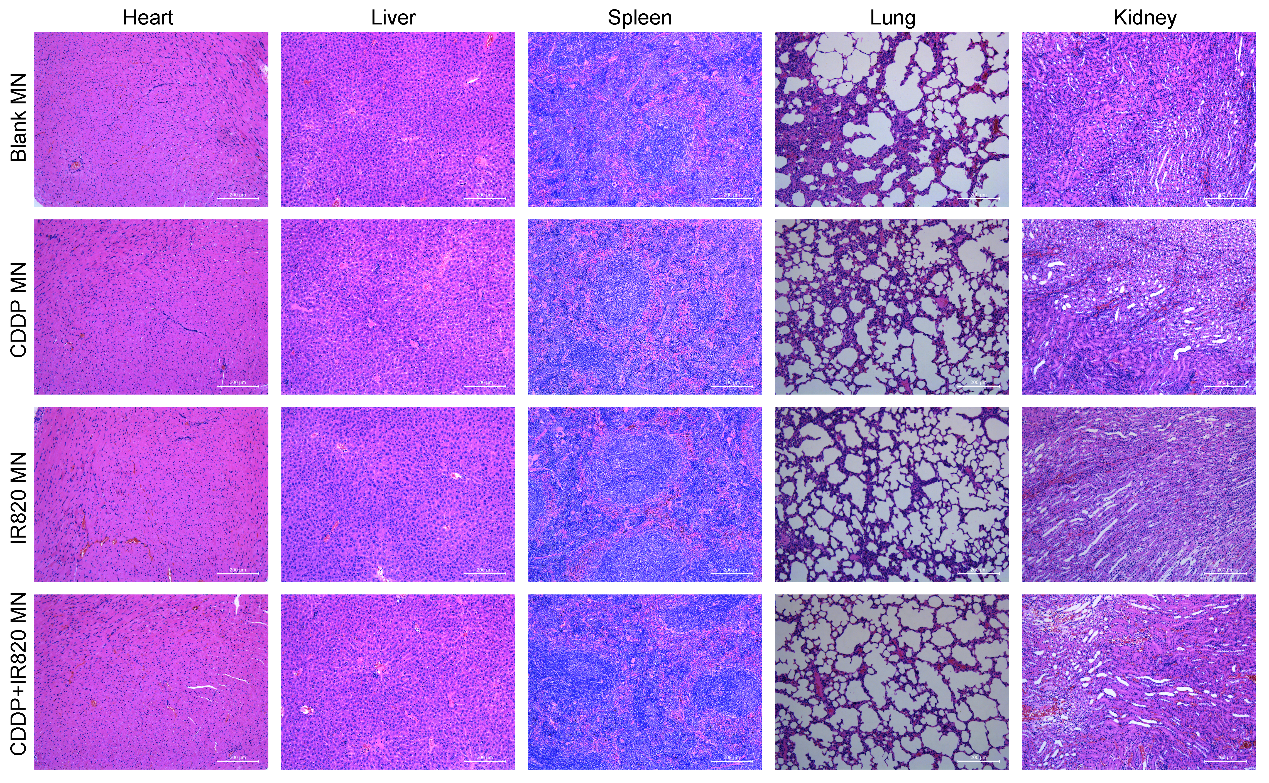


Fig. S1 H & E staining of the main organs after the MN patches treatment (the scale bar represents 200 µm).


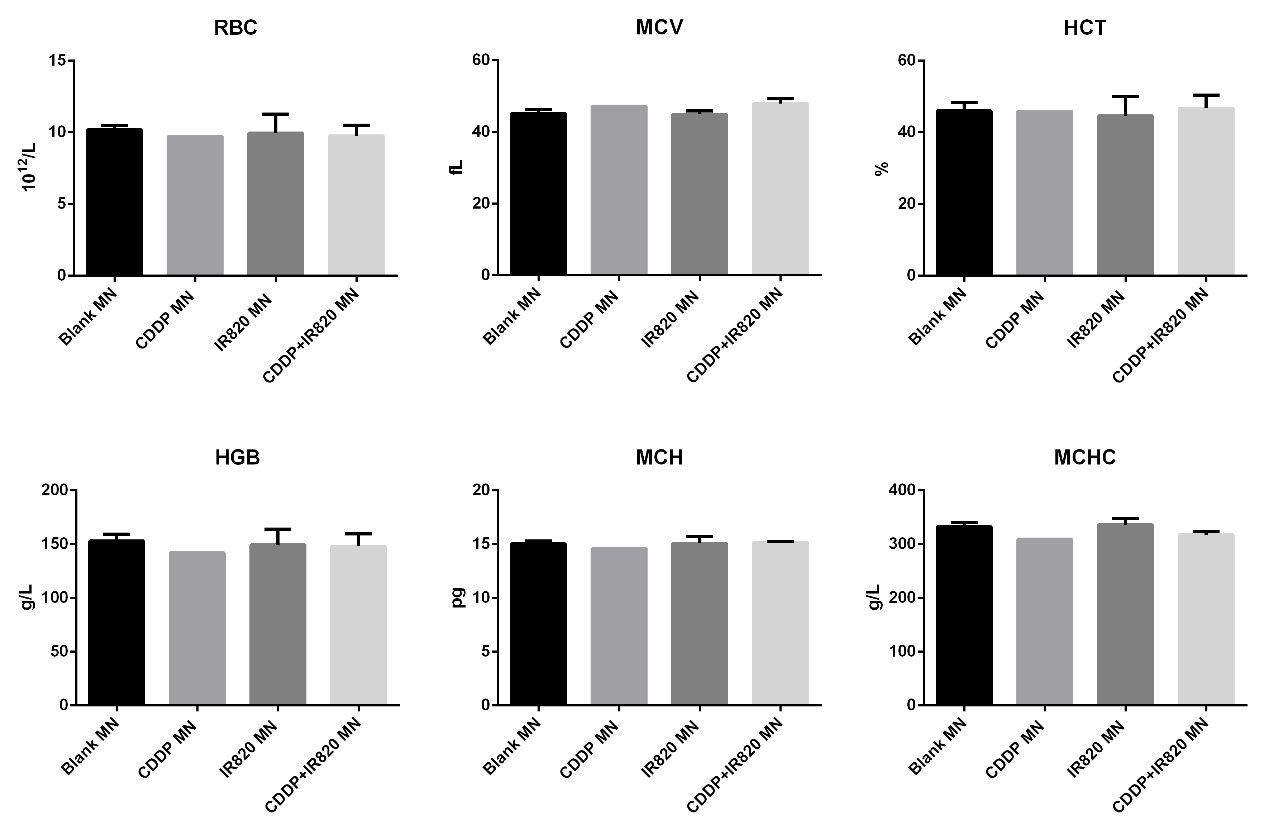


Fig. S2 Blood analysis of the MN patches treated mice.


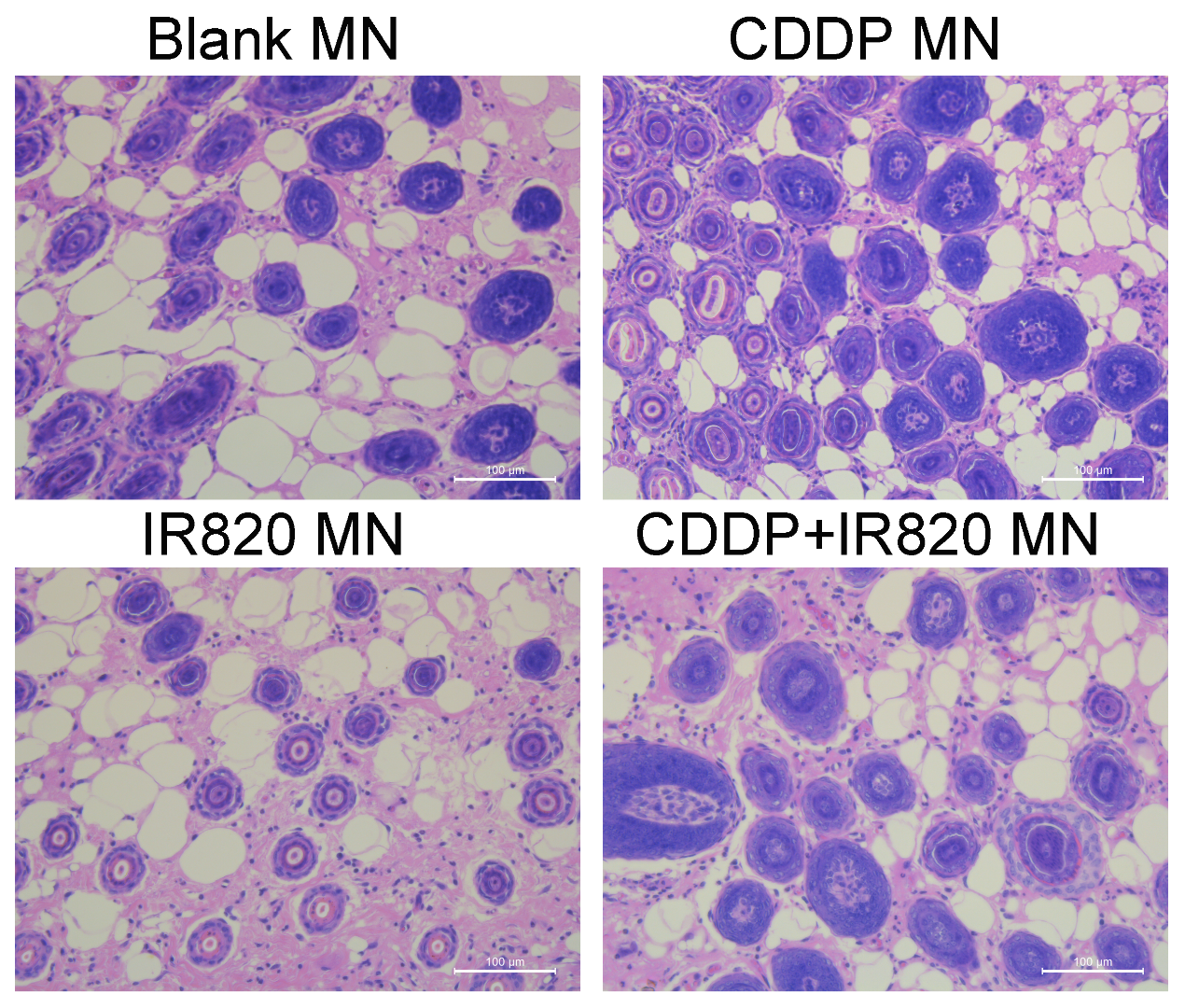


Fig. S3 H & E images of the skin tissues after the MN and/or 808 nm laser treatment (the scale bar represents 100 µm).
